# An embryological perspective on the early arthropod fossil record

**DOI:** 10.1186/s12862-015-0566-z

**Published:** 2015-12-18

**Authors:** Ariel D. Chipman

**Affiliations:** The Department of Ecology, Evolution and Behavior, The Hebrew University of Jerusalem, Edmond J. Safra Campus, Givat Ram 91904, Jerusalem, Israel; The Department of Paleobiology, The Smithsonian Museum of Natural History, Washington, DC USA

**Keywords:** Arthropods, Paleontology, Evo-devo, Embryology, Germband, Cambrian Explosion

## Abstract

**Background:**

Our understanding of the early evolution of the arthropod body plan has recently improved significantly through advances in phylogeny and developmental biology and through new interpretations of the fossil record. However, there has been limited effort to synthesize data from these different sources. Bringing an embryological perspective into the fossil record is a useful way to integrate knowledge from different disciplines into a single coherent view of arthropod evolution.

**Results:**

I have used current knowledge on the development of extant arthropods, together with published descriptions of fossils, to reconstruct the germband stages of a series of key taxa leading from the arthropod lower stem group to crown group taxa. These reconstruction highlight the main evolutionary transitions that have occurred during early arthropod evolution, provide new insights into the types of mechanisms that could have been active and suggest new questions and research directions.

**Conclusions:**

The reconstructions suggest several novel homology hypotheses – e.g. the lower stem group head shield and head capsules in the crown group are all hypothesized to derive from the embryonic head lobes. The homology of anterior segments in different groups is resolved consistently. The transition between “lower-stem” and “upper-stem” arthropods is highlighted as a major transition with a concentration of novelties and innovations, suggesting a gap in the fossil record. A close relationship between chelicerates and megacheirans is supported by the embryonic reconstructions, and I suggest that the depth of the mandibulate-chelicerate split should be reexamined.

## Background

The evolution of the arthropod body plan has been debated extensively from the earliest days of evolutionary morphology. While this debate is still very much ongoing, a consensus is starting to emerge on many of the key questions. A better-resolved phylogenetic framework, recent advances in the understanding of arthropod development, and numerous new fossils from early in the arthropod evolutionary history have combined to give a clearer understanding of the stepwise evolution of arthropod body plans from the earliest arthropod stem.

With a phylogenetic tree at hand, reconstructing the evolutionary history of the body plan requires mapping apomorphies on this tree, be they character transitions or novelties. Frequently, these apomorphies are based on adult morphology, in what has been critically called the “adultocentric” view of evolution [1]. However, the source of most morphological transitions and novelties lies in changes in ontogeny. In this paper, I bring a developmental perspective to early arthropod evolution and interpret the major events in the evolution of the arthropod body plan from the point of view of changes in early development. To this end, I use the understanding gleaned from the analysis of development of extant taxa to suggest possible processes involved in the development of fossil taxa.

### Phylogenies

The most important factor in reconstructing morphological evolution is having a reliable and well-resolved phylogenetic framework. Achieving an accepted phylogeny had been one of the most contentious issues in arthropod evolution for many decades. This issue is in fact made up of two separate questions. One is establishing the relationships among extant arthropod taxa, and the other is the larger phylogenetic framework of Arthropoda sensu *lato*, including stem groups and other fossil taxa.

Study of the phylogeny of extant arthropods has improved significantly in the past few years with the addition of large-scale molecular datasets [2–5], and it is now becoming increasingly likely that the phylogenetic relationships among extant arthropods have been correctly resolved. The consensus tree includes Hexapoda (including Insecta) as part of Pancrustacea, with the crustaceans being paraphyletic with respect to hexapods. Pancrustacea in turn is allied with Myriapoda under Mandibulata. Chelicerata forms a separate branch, which includes Arachnida and Xiphosura as sister taxa (jointly known as Euchelicerata), with Pycnogonida as a sister group to them. Some molecular phylogenies dispute the placement of Myriapoda within Mandibulata, and ally them instead with Chelicerata [3, 6]. However, this placement is believed by most workers in the field to be the result of methodological artifacts [4]. Two non-arthropod phyla are included with the arthropods under Panarthropoda: Tardigrada and Onychophora. It has not been as easy to reach a consensus on which of these two is more closely related to Arthropoda, although the bulk of the data point to Onychophora as the arthropods’ closest living sister group [7].

The phylogenetic placement of fossil forms within the larger arthropod tree remains controversial, although here also several key nodes have been recently resolved in a way that seems consistent with multiple sources of data and may soon reach a consensus. The placement of others has been more difficult and even the monophyly of several classic groupings (e.g. anomalocaridids, great appendage arthropods) has been challenged. Nonetheless, phylogenetic analyses of the last few years are converging on an accepted tree.

**Fig. 1:**
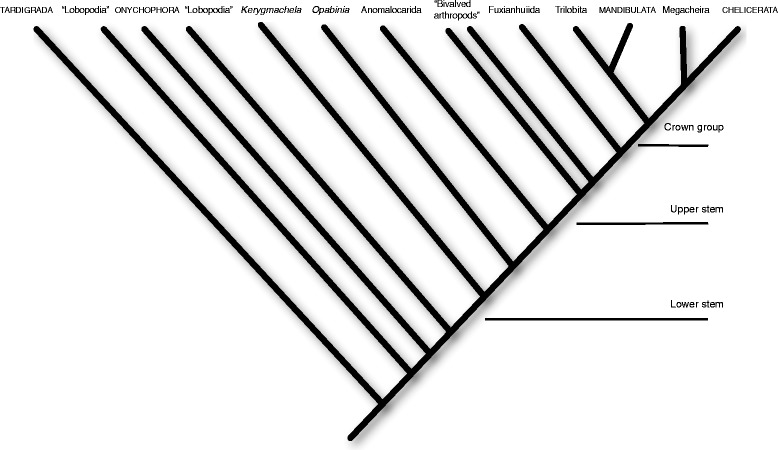
The phylogenetic hypothesis of Panarthropoda used for this work. The phylogeny is compiled and simplified from several sources, most notably [8], with some modifications. Node names in italics are individual species. Names in capitals are extant taxa. All others are fossil taxa. The phylogeny is divided into three main groups: lower stem taxa, upper stem taxa and crown group Arthropoda. Some members of the “Lobopodia” probably belong in the arthropod lower stem (e.g., *Jianshanopodia*, *Megadictyon*), but due to the lack of resolution in that group, and because they are outside the scope of this analysis, the stem group is marked as beginning with *Kerygmachela*

The phylogenetic framework used here (Fig. 1) is based on the thorough morphology-based analysis of arthropod phylogeny, combining fossil and extant taxa, by Legg et al. [8], with some modifications. It is not my aim to enter the debate between conflicting phylogenies, or to favor one phylogeny over another. Rather, this phylogeny is taken as a working hypothesis, based on what seems like the best current consensus. Where a different topology would have differing consequences for my analysis, these are specifically discussed.

The backbone of this phylogeny includes Tardigrada as the earliest branching group and sister to all others, followed by a paraphyletic series of lobopodians. Onychophora are placed within this paraphyletic grade. Next up the tree are the gilled lobopodians, exemplified by *Kerygmachela*, followed by the well-known *Opabinia*. The anomalocaridids, properly known as Radiodonta, are the next closest to crown arthropods. The traditional Anomalocaridida may not be monophyletic [9] and includes taxa that some authors remove from Radiodonta and place with the more crownward megacheirans (e.g. *Parapeytoia*) [8, 10]. Rather than exploring this debate, in the current analysis, I circumvent the problem by dealing only with the anomalocaridids that are universally accepted to belong to Radiodonta, and treat them as monophyletic. A diverse group of “bivalved” arthropods follows, including *Isoxys* and *Canadaspis* among many others, and these are again seen as a paraphyletic grade. The closest sister group to the crown group arthropods is the monophyletic Fuxianhuiida. In Legg et al.’s [8] phylogeny there is an additional outgroup before crown arthropods, the short great-appendage arthropods, or megacheirans. However, many authors consider these to be crown-group arthropods, closely related to – or even members of – Chelicerata [11–14]. I follow this view and include Megacheira as a sister group to Chelicerata. The only other fossil crown-group arthropods dealt with in the current analysis are the trilobites. Trilobita is universally agreed to be monophyletic, though its position relative to other crown taxa is still debated. Legg et al.[8] place trilobites under Artiopoda together with Chelicerata and several other taxa. However, they are alternatively allied with the Mandibulata [15], and their morphology is easier to accommodate within such a framework, which I adopt here.

### Homologies

The homology of different arthropod body regions, and especially those in the anterior, has been the second major debate within arthropod biology for many years. Like the debate on phylogeny, this debate is also reaching a consensus, and many opposing points of view that seemed intractable only a decade ago have been resolved based on new data from comparative embryology, gene expression data and neuro-morphology.

It is now broadly accepted that the anterior segments in all arthropods and in onychophorans can be aligned [15–18]. This paper accepts the following assumptions of homology. As with the phylogeny, this is a working hypothesis, based on a consensus view, and I make no explicit claims about their correctness. The anterior-most or protocerebral segment includes the eyes, and its appendage is the antenna of onychophorans, the frontal raptorial appendage of gilled lobopods and anomalocarids and the hypostome-labrum of crown-group arthropods (see Discussion for the problematic definition of this segment and its subdivision). The second or deutocerebral appendage includes the jaw of onychophorans, the raptorial appendage of megacheirans, the antenna of insects and myriapods and the antennule of crustaceans, and the chelicera of chelicerates. The third or tritocerebral segment includes the slime papilla of onychophorans, the antenna of crustaceans, the intercalary segment of insects and myriapods and the pedipalps of most chelicerates or first walking limb of xiphosurans. The fourth segment carries the first walking limbs in onychophorans and chelicerates and the mandibles of mandibulates. Consecutive posterior segments to this carry walking legs or modified feeding limbs of some sort or other.

In this analysis, I also assume the homology of all paired eyes and of all medial eyes, where present. I assume that the dorsal flaps found in gilled lobopods and anomalocaridids are all homologous, and that jointed limbs and lobopods are homologous to each other. A recent report [19] has identified two sets of flaps in anomalocaridids, and it suggests that “ventral flaps are homologous with lobopodous walking limbs and the endopod of the euarthropod biramous limb, whereas the dorsal flaps and associated setal blades are homologous with the flaps of gilled lobopodians” (p. 77). While it is too early to embrace this idea (or to reject it), I include it my discussion in the relevant section.

### The early germband as the arthropod phylotypic stage

Most extant arthropod embryos pass through a germband stage [20, 21]. This stage is highly conserved morphologically, and can be seen as the phylotypic stage for arthropods. To be more precise, the germband undergoes numerous changes in its development, and the stage is probably better seen as a “phylotypic period”[22]. Indeed, in the early germband stage, many arthropod embryos are so similar as to be nearly indistinguishable (personal observation). During this stage the embryonic axis is fully determined, and is divided into segments that can first be identified by a conserved set of gene expression patterns, and shortly afterwards by overt morphological segmentation [23]. The germband includes a distinct anterior area that is usually broader than the rest of the germband and is referred to as the head lobes. Head lobes are found even in chelicerate embryos, which do not have a distinct head as adults. The embryonic axis bifurcates in the head lobes, which in mandibulates are composed of 3–4 segments that are never morphologically distinct, but can be identified based on segmental gene expression.

As the germband develops, limb buds appear on segments that carry limbs in the adult/larval phase [24]. These limbs extend and become differentiated, at which point – the late germband – embryos of different taxa start diverging from each other significantly and displaying taxon specific characters.

An important caveat to defining the germband as the phylotypic stage is that not all arthropods display it. Many arthropods, most notably the majority of crustacean groups, hatch as a head larva or nauplius, which has only three anterior segments that develop and differentiate fully before the formation of any posterior segments. The question of whether the head larva is plesiomorphic for arthropods is yet another hotly debated subject, and will be dealt with separately in the Discussion.

### The late germband as a representation of the body plan

The typical body plan of arthropod higher-level taxa (equivalent to Linnaean classes and orders) is first manifested at the late germband stage. Despite the morphological similarities alluded to above in the early germband stage, throughout the late germband stage the body plan diversifies, and taxon specific characters are first seen. The number and position of appendages, differentiation into distinct tagmata and differences in the number and organization of the segments in the head lobes all become apparent throughout the later germband stage.

This organization of germband morphology represents the structure that is typical of arthropod taxa at the so-called ordinal or class level. Germbands of insect embryos all have the same organization of the head lobes, the same number and location of limb buds and the same total number of segments, as is typical for Insecta. Similarly, all spider germband stage embryos have the same arrangement. It can be said that the typical body plan of these taxa is first manifested at the late germband stage. Furthermore, characters that are indicative of lower level taxonomic groupings are not yet evident in the late germband. More specific specializations of the limbs in insects, number and arrangement of eyes or spinnerets in spiders, or differentiation of limb types in crustaceans only become apparent much later. This observation is of course not new, and was stated by Von Baer in 1828 as his first law of development.

These characteristics thus make the germband stage, spanning the period from the first formation of phylotypic structures to the manifestation of higher taxonomic level body plan, a crucial stage to study for understanding the evolution of the arthropod body plan.

### Hypothetical embryology of extinct taxa

What is true for extant arthropods is likely to have been true throughout the evolution of arthropods and their relatives. Just as the body plan is manifested during the germband stage of living taxa, so we can assume that the germband of fossil organisms represented the earliest development of their body plan, and stem group taxa had identifiable structures in the germband stages of their development. In this analysis I suggest hypothetical reconstructions of germband stages of fossil taxa, based on what we know of the development of extant species (see Methods). This is naturally speculative, and should be best seen as a thought experiment rather than as a series of testable hypotheses. However, by representing fossil taxa as embryos we have a simplified and distilled version of their body plans. These representations offer new insights into the transformations and novelties that were involved in the attainment of the arthropod body plan during the early evolution of this most diverse and species rich phylum.

## Results and discussion

The core of this exercise in fossil embryology is a reconstruction of germband stage embryos of a series of fossil taxa. I have drawn sketches of what the embryos may have looked like at two stages for a group of selected taxa, representing key branches on the arthropod phylogenetic tree, and plotted additional relevant data on these sketches.

### Simplifying assumption

The germband is a dynamic, complex, three dimensional structure. The reconstructions presented here include a number of simplifications. The first simplification is that all germbands are represented as conceptually, “dissected” from yolk, chorion and other egg structures, and flattened. There is also no attempt to present the germbands with any type of scaling, and they are all drawn at the same size. The size of the egg, the amount of yolk and the arrangement of the germband on or in the yolk are highly variable even over close phylogenetic distances, and are usually irrelevant to the actual morphology. The second simplification is that the germbands are shown as internally synchronized. In reality, a germband shows a time axis along its antero-posterior axis, with anterior segments being more mature than posterior segments. This inter-segmental difference can be extreme in the case of indirect development, where some posterior segments are patterned post-hatching or even in successive molts (as in anamorphic centipedes [25, 26] or in trilobites [27]). In the current reconstructions all segments are shown to be the same age and all patterned together in the germband.

In an effort to keep this analysis within a manageable size, there are several important questions that I do not discuss, even though interesting insights might be garnered from thinking about them in an embryological context. These include the question of posterior growth (which will be dealt with separately in a future manuscript); The evolution of different types of eyes (this has been dealt with in detail by Strausfeld et al. [28]); The evolution of the nervous system and the brain; and the transition between uniramous and biramous limbs (see recent discussion by Edgecombe [29]).

### Taxonomic representation

For the sake of this analysis, the reconstructions are mostly done at the level of super-specific taxa – usually at or around the generic or family level. The exceptions are in cases where only a single fossil species is known for a higher-level taxon. In other cases, the reconstruction is of an unknown ancestor of a crown group taxon.

The analysis starts at the arthropod stem, with *Kerygmachela kierkegardii* and *Opabinia regalis*, each representing unique species. Next up the tree are the anomalocaridids, treated together as a single reconstruction, and based mostly on a generalized *Anomalocaris*. These three nodes are referred to together as “lower stem” arthropods (sensu [30]).

The “upper stem” begins with the bivalved arthropods. This is clearly a paraphyletic and possibly a polyphyletic grouping with highly variable morphology. It is represented with a single reconstruction, based mostly on *Branchiocaris pretiosa*. This is followed by Fuxianhuiida, based mostly on *Fuxianhuia protensa*.

Crown-group arthropods include a reconstruction of a hypothetical crown-group mandibulate ancestor, based on data from numerous extant taxa (insects, crustaceans and the centipede *Strigamia maritima*), and a hypothetical crown-group chelicerate ancestor based mostly on *Parasteatoda tepidariorum* and *Phalangium opilio* with added details from *Limulus polyphemus*. Fossil groups within the crown group include Trilobita, which is not reconstructed here, and Megacheira represented by a single reconstruction based mostly on *Leanchoilia* spp. and *Yohoia tenuis.*

### Kerygmachela

**Fig. 2:**
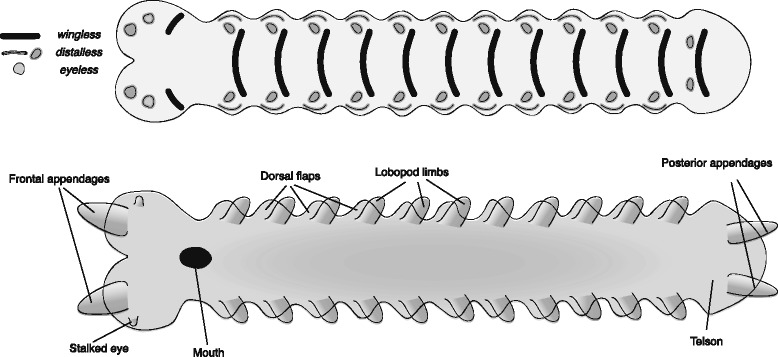
Reconstructed germband of *Kerygmachela kierkegardii*. The upper panel represents an early stage germband with the expression of select marker genes mapped onto it. The lower panel represents a late stage germband, annotated with the adult fate of the main embryonic features. Note distinct *distalless* domains for the dorsal flaps and the lobopods in the upper panel

The reconstructed *Kerygmachela kierkegardii* germband (Fig. 2) is based on an onychophoran germband [31–34] with relevant modifications relating to the identity of limbs and the addition of flaps. The reconstruction represents the first steps towards the attainment of the arthropod body plan from the ancestral lobopod/onychophoran-like body plan [35].

One of the defining features of *K. kierkegardii* and many other lower-stem arthropods are the dorso-lateral flaps. These have been interpreted as homologous to the outer branch of the arthropod limb – the exopod (in anomalocaridids [19] and by extension, presumably for other lower-stem groups) or as gill-bearing body-wall outgrowths [35, 36]. These flaps are found in addition to lobopod limbs, positioned more ventrally. In the reconstruction of the early germband, I place the anlage of the flaps as lateral segmental bulges, expressing *distalless*, and the anlagen of the lobopods more medially (corresponding to a more ventral position post-embryonically), also expressing *distalless.*

The anterior raptorial appendages are of protocerebral origin, and I place their anlagen in the anterior tip of the early germband, extending forward from the protocerebral lobes at later stages. Rearward facing appendages are reconstructed on the posteriormost segment, providing the anlagen of the posterior spines. The stalked eyes of *K. kierkegardii* probably represent a primitive state [28], and probably shared some embryological characteristics with limbs. To indicate this, I have reconstructed them as emerging from a region that expresses *distalless* as well as *eyeless*. The mouth is reconstructed between the protocerebral and deutocerebral segment, as it is in onychophorans. However, this position of the mouth is tentative, as the ventral shift of the mouth may be convergent between arthropods and onychophorans, and Budd reconstructs the mouth of *K. kierkegardii* as terminal [35].

### Opabinia

**Fig. 3:**
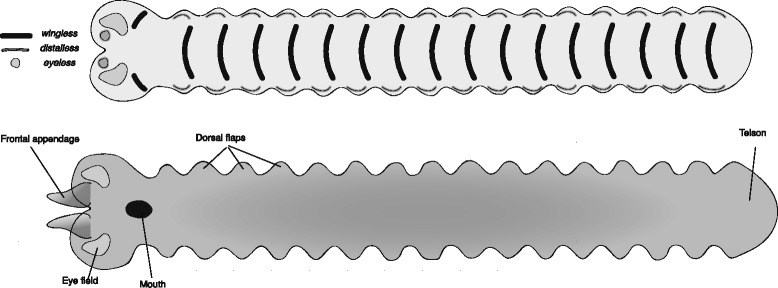
Reconstructed germband of *Opabinia regalis*. The upper panel represents an early stage germband with the expression of select marker genes mapped onto it. The lower panel represents a late stage germband, annotated with the adult fate of the main embryonic features. Note the close antero-medial position of the paired frontal appendage anlagen. These are assumed to fuse at dorsal closure to give a single appendage. The origin of the eyes has not been reconstructed in detail, and a broad “eye field” is shown instead

The fossil *Opabinia regalis* from the Burgess Shale is the “poster-child” of the weird and wonderful view of the Cambrian world [37]. Despite its many seemingly bizarre characteristics, its germband stage embryos can be reconstructed as fairly simple (Fig. 3), and it is in fact very similar to that reconstructed for *Kerygmachela*, due to their sharing of many plesiomorphic characters.

There is an ongoing debate as to whether *O. regalis* had lobopods as well as dorso-lateral flaps [38, 39], or whether the putative lobopods are actually gut diverticula [37, 40]. In this reconstruction I have accepted the latter approach. I have not drawn limb buds and have not added *distalless* domains for the lobopods, but only for the dorso- lateral flaps. However, if the former interpretation, in which *O. regalis* had lobopods as well, turns out to be correct, the reconstruction would include additional *distalless* domains and limb buds similar to those portrayed for *K. kierkegardii*.

The most intriguing aspect of *O. regalis’* morphology is probably the single flexible frontal appendage. The only reasonable interpretation of this appendage is as a fusion of the paired protocerebral appendages found in *K. kierkegardii* and in the more crownward anomalocaridids [41]. Therefore, I have reconstructed the early germband with paired anterior *distalless* domains, and a later pair of limb buds, but I have positioned these more medially than in *K. kierkegardii* to allow them to fuse in later development, following dorsal closure.

Equally intriguing are the five stalked eyes of *O. regalis*. The embryological origin of these eyes is not clear, and I have reconstructed a broad “eye domain”, without specific details. Many of the intriguing characters of *O. regalis* (single anterior appendage, five dorsal eyes) can be seen as autapomorphic, and therefore not directly relevant to the gradual evolution of the arthropod body plan. Their unique appearance in this taxon makes it difficult to interpret their evolution and reconstruct the transitions that led to them. The loss of lobopods can either be seen as autapomorphic, if anomalocaridid ventral flaps are seen as homologous to lobopods, or as a synapomorphy uniting *Opabinia* and all other higher groups.

### Anomalocaridida

**Fig. 4:**
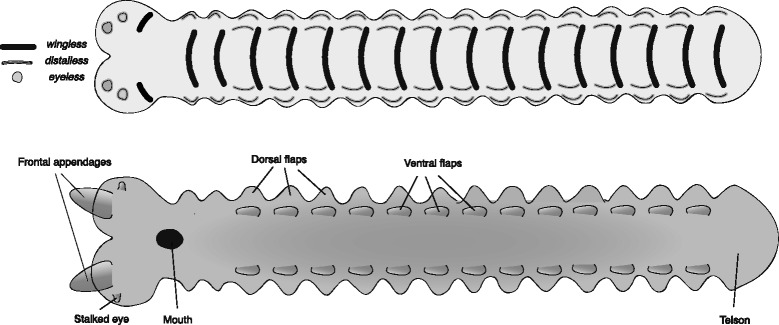
Reconstructed germband of Anomalocaridida, based mainly on *Anomalocaris canadensis*. The upper panel represents an early stage germband with the expression of select marker genes mapped onto it. The lower panel represents a late stage germband, annotated with the adult fate of the main embryonic features. The reconstruction includes two pairs of flaps: dorsal flaps and ventro-lateral flaps

The anomalocaridids (also known as Radiodonta) are a large and diverse group, with over 20 identified species [8, 19, 42]. They vary greatly in size and in dietary adaptations, but share a conserved body plan. The reconstructed germband (Fig. 4) is based on data from recent descriptions of several species [19, 42, 43], but follows *Anomalocaris canadensis* in specific details [44].

According to most descriptions, anomalocaridids had a single row of paired dorsal flaps, and no lobopods, making the reconstruction of the trunk region of the germband very similar to that of *O. regalis.* However, a recent report [19] argues that most anomalocaridids actually had two sets of paired flaps, a ventral row and a dorsal row. Furthermore, it has been suggested that the ventral flaps represent a vestige of the missing lobopods and are thus homologous to them [19, 29]. In the reconstruction of the anomalocaridid germband I follow this latest interpretation. I reconstruct the dorsal flap anlagen similar to those of *K. kierkegardii* and *O. regalis*. I reconstruct the ventral flap as a narrow band of *distalless* expression in the early germband, and as a narrow and long bulge in the later germband, slightly medial to the dorsal flap anlagen. I suggest that during dorsal closure, the two structures drift further apart as the lateral margins of the germband are stretched dorsally. However, it is conceivable that over evolutionary time, these two domains could have become fused, to form the ancestral biramous limb, as suggested by Van Roy et al. [19].

The raptorial frontal appendages are protocerebral and emerge from the anterior of the germband, as in the two previous taxa discussed. Notably, the appendages are segmented, representing the earliest cases of jointed appendages (contra [45]) in the arthropod stem group, and the putative precursor of the arthropodal limb. However, based on extant arthropods, limb segmentation is not evident at the germband stages portrayed here, so this is not indicated in the sketched reconstructions.

### “Bivalved” arthropods

**Fig. 5:**
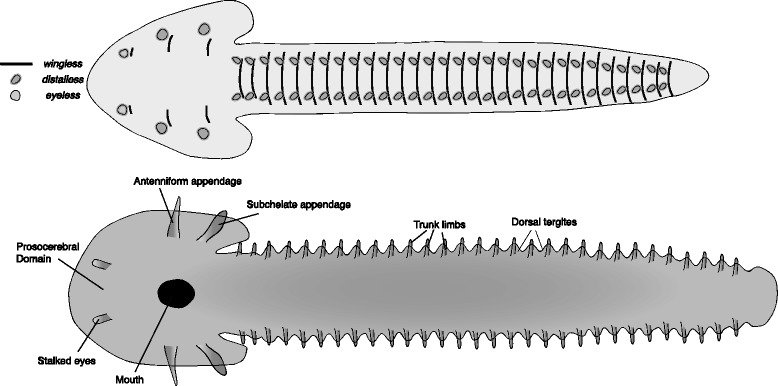
Reconstructed germband of the bivalved arthropods, based mainly on *Branchiocaris pretiosa*. The upper panel represents an early stage germband with the expression of select marker genes mapped onto it. The lower panel represents a late stage germband, annotated with the adult fate of the main embryonic features. The large anterior lobe forms the origin of the bivalved shield following dorsal closure. The protocerebral segment in this group and in all crownward groups, is divided into two domains, the prosocerebral domain, which is medial and gives rise to the anterior sclerite [49, 53], and the archicerebrum, which lies lateral to it and gives rise to the eye. The border between the two is not marked

The diverse assemblage of bivalved arthropods is almost definitely paraphyletic and possibly even polyphyletic. The discussion below is based on descriptions of several different bivalved arthropods [46–50], although the pictured reconstruction (Fig. 5) is based on *Branchiocaris pretiosa* [49].

The transition between the lower-stem and upper-stem arthropods, as exemplified by the differences between anomalocaridids and bivalved arthropods, is a major one, including numerous novelties and character state transformations. Some of these may have occurred within the bivalved arthropods, but it seems that most of them took place within a series of forms, basal to the bivalved group, that have unfortunately left no trace in the fossil record (see [30] for a detailed discussion). The internal relationships among the bivalved arthropods are unclear, and descriptions of different species include apparent contradictions that make homologies difficult to ascertain. The following discussion is an attempt at a unified description of the embryonic transformations that occurred during this pivotal phase in arthropod evolution. It is not fully consistent with all published descriptions and phylogenies, and may prompt a re-appraisal of some interpretations.

In contrast with the anomalocaridids, bivalved arthropods normally have multiple head appendages and thus a head composed of more than one segment. They have articulated limbs post-orally. They have a clearly arthrodized body (i.e. a body made of stiff segmental cuticular elements, connected by flexible membranes). In addition, the eyes are oriented antero-ventrally, unlike the dorsally oriented eyes of anomalocaridids and gilled lobopodians.

The defining feature shared by all bivalved arthropods (though possibly convergent) is a bivalved carapace or shield covering the anterior of the body. The shield is usually understood to be hinged dorsally and to connect to the trunk along the dorsal margin of the anterior body. To reconstruct the embryonic origin of the shield I have turned to extant arthropods with analogous structures, namely the ostracods. Relatively little is known about the early development of ostracods. The two main descriptions are of *Vargula hildendorfii* [51] and of *Manawa staceyii* [52]. Fig. 3b in Wakayama (2007) [51] seems to show the rudiment of the shield as being contiguous with the head lobes of the germband. Swanson (1989) [52] shows the shield as starting univalved and then splitting. Following this analogy, I reconstruct the germband embryo of bivalved arthropods as having very large anterior lobes, which will fuse dorsally during dorsal closure and give rise to the anterior shield, and develop a dorsal ridge/fold later in development. I suggest that these lobes are a novel embryonic structure that arose in the lineage leading to some or all of the bivalved arthropods. I raise the possibility that these lobes later formed the basis for external head structure, thus identifying the bivalved shield as an early homolog of the crown group head.

*B. pretiosa*, like several other bivalved taxa, is interpreted as having an elongated sensory (antenniform) appendage and a subchelate feeding appendage. Based on their location and based on interpreted homology with anterior appendages of fuxianhuiids, these appendages can be assigned to the deutocerebral and tritocerebral segments respectively. This interpretation has been strengthened by the identification of a conserved anterior sclerite, which represents the protocerebral segment, and lies clearly anterior to the two appendages [49, 53]. The reconstructed germband of the bivalved arthropods thus includes two limb buds lying within the extended anterior lobes, in addition to an anterior eye field with a stalked-eye bud. Trunk limbs are reconstructed along the germband, posterior to the anterior lobes.

The transition from lower-stem to upper-stem arthropods must have involved a complex change in the structure of the anterior germband. As suggested above, novel anterior lobes appeared, and formed a head shield. The appearance of these lobes defined a novel anterior domain in the germband – the first vestige of a multi-segmented head. Within this domain, the anterior appendage was lost, but two posterior appendages appeared, presumably recruiting the genetic networks for making a segmented limb from the lost anterior limb. These two novel limbs further differentiated to give two distinct morphologies. The anteriormost structure of the germband, homologous to the appendage-bearing domain of the lower-stem taxa’s anterior appendage, retreats posteriorly, thus splitting the anterior of the embryonic axis. This structure, sometimes known as the prosocerebrum (or pre-protocerebrum), is part of the protocerebral segment, together with the eye bearing domain, the archicerebrum [43]. In the posterior germband the ancestral lobopod-type limbs also recruited the networks for making segmental limbs (possibly following the fusion of two sets of lateral flaps).

The preceding description is confounded by interpretations of the abundant bivalved arthropod *Isoxys* and its close relatives (e.g. *Surusicaris* [46]). This group includes numerous species, distinguished by variable morphologies of their carapace [48]. Recent descriptions of isoxyid fossils include a single anterior raptorial appendage, which is variably identified as either protocerebral or deutocerebral [46, 48]. If the protocerebral interpretation of the raptorial appendage is correct, it would place isoxyids as a transitional group between the lower-stem and upper-stem, representing the appearance of the shield (and presumably of anterior lobes in the germband) before the appearance of the antenniform deutocerebral and subchelate tritocerebral appendages, and possibly before the loss of the protocerebral appendage, but later than the recruitment of the segmented limb patterning network to the trunk limbs. The interpretation of some of the isoxyid fossils has been disputed [30], and it is possible that indeed the raptorial appendages of *Isoxys* and its relatives were tritocerebral and there was an additional highly reduced deutocerebral antenniform appendage that has not been identified.

### Fuxianhuiids

**Fig. 6:**
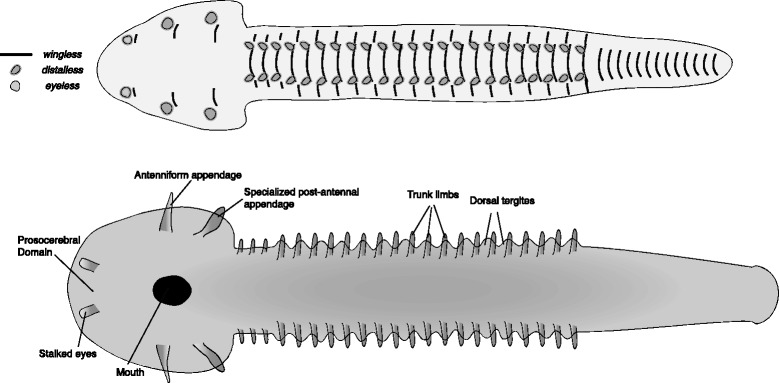
Reconstructed germband of Fuxianhuiida, based on *Fuxianhuia protensa*. The upper panel represents an early stage germband with the expression of select marker genes mapped onto it. The lower panel represents a late stage germband, annotated with the adult fate of the main embryonic features. Note the mismatch between dorsal tergites and limbs, both in the gene expression at the early stage represented in the upper panel and in the limb buds and tergites in the lower panel. The anterior lobes are smaller than in the bivalved taxa, and give rise to the head shield

The fuxianhuiids are less diverse than the bivalved arthropods. The reconstruction presented here (Fig. 6) is based on *Fuxianhuia protensa*, possibly the best known of stem group arthropods due to a number of exceptionally preserved fossils displaying internal anatomy [54–56]. In terms of the reconstructed germband, fuxianhuiids are not significantly different from the bivalved arthropods. Their head shield is significantly smaller than the shield of bivalved arthropods, and I have accordingly portrayed the anterior lobes as smaller. The antenniform appendages are homologous to those of bivalved arthropods and have been reconstructed similarly in the germband. The “specialized post-antennal appendage” (SPA) [56] is likewise homologous to the raptorial appendage of the bivalved group, and has been reconstructed in the same way.

Specific features of *F. protensa* include three small anterior trunk appendages, presumably adapted for manipulating food in a manner similar to that of maxillipeds in some crustaceans. These are indicated as smaller limb buds in the late germband. In addition, there is a mismatch between dorsal segment boundaries and individual limbs, similar to what is seen in *Glomeris marginata* [57], and in several other examples. As in *Glomeris* I have indicated separate segmental gene expression for the dorsal (lateral) and ventral (medial) domains in the early germband. The posterior of the animal includes a series of limbless segments. The different types of limbs in the trunk and the existence of a limbless domain is the first example of true tagmatization – more fully developed in crown group arthropods.

### Crown group Mandibulata

**Fig. 7:**
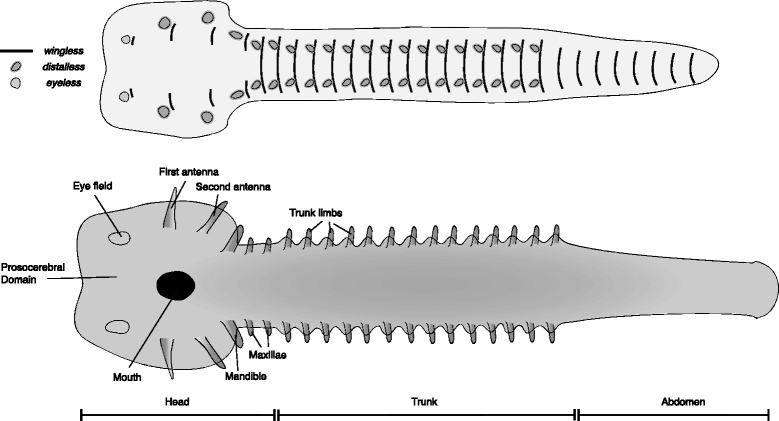
Reconstructed germband of the common ancestor of crown group Mandibulata, based on numerous extant taxa. The upper panel represents an early stage germband with the expression of select marker genes mapped onto it. The lower panel represents a late stage germband, annotated with the adult fate of the main embryonic features. The specific reconstruction includes three distinct tagmata – the head, which includes three post antennal segments; the anterior/trunk tagma (pereon/thorax-like) with limbed segments; and the posterior/abdominal tagma (pleon-like) with limbless segments

The hypothetical embryo of the ancestor of the mandibulate crown group is reconstructed based on a number of well-studied taxa. However, it should be noted that these taxa represent a sampling of relatively derived taxa, and do not represent the full diversity of developmental modes. As discussed above (Simplifying Assumptions) and below (Direct vs. Indirect Development), it is possible that the actual mandibulate ancestor had indirect development, with a biphasic (or multi-phase) extension of the germband. The reconstructions presented in Fig. 7 are based on data from a number of insect embryos (including *Tribolium castaneum* [58–61]*, Oncopeltus fasciatus* [62–65] *and Gryllus bimaculatus* [66–68]), the embryo of the centipede *Strigamia maritima* [69, 70] and embryos of the amphipod *Parhyale hawaiensis* [71, 72], the isopod *Porcellio scaber* [73, 74] and the branchiopod *Daphnia magna* [75].

The reconstructed ancestral mandibulate embryo has distinct head lobes, which represent the anlage of four head segments. The embryonic axis bifurcates in the head lobes, with the four anterior segments being formed as hemi-segments on either side of an anterior median region [60, 76]. The protocerebral segment has no obvious embryonic appendage. The eyes are unstalked and represented with no *distalless* domain. The deutocerebral and tritocerebral segments both carry elongate appendages. The appendageless tritocerebral segment in insects and myriapods is a convergent autapomorphy in both clades. Numerous Orsten type fossil species, belonging to stem-group Mandibulata, carry two pre-mandibular appendages [77], an antennule and an antenna (though it is not always antenniform) and this is thus believed to be the ancestral form for mandibulates. The fourth segment bears a gnathobasic feeding appendage – the eponymous mandible – a defining synapomorphy for the clade. The anlage of this segment is at the base of the head lobes in some extant taxa, or just posterior to it in others, and is either split or not split respectively. I have reconstructed it as split, but this is an arbitrary choice, and the ancestral state is equally likely to have been un-split. The following two segments also bear appendages (which function in feeding in most – but not all – crown mandibulates). I have reconstructed the embryo with two distinct tagmata; an anterior trunk with limbs – similar to the malacostracan pereon, or insect thorax – and a limbless posterior tagma – similar to the malacostracan pleon or insect abdomen (the number of segments in both is arbitrary). In some lineages, most notably insects, but perhaps others as well, the formation of the head capsule in later development involves a bending of the anteriormost segments [78], bringing the antennae and eyes to a dorsal position in the head, and incorporating the gnathal segments into the head capsule. In order to maintain a ventral position of the mouth, the stomodeum retreats from its deutocerebral position and is found at the level of the tritocerebral segment.

### Trilobites

I do not represent a reconstructed germband for the trilobites. This is largely due to the fact that unlike all other fossil taxa, a great deal is known about trilobite ontogeny and it is clear that they developed anamorphically without a segmented germband stage [79, 80]. Thus, it seems pointless to reconstruct a stage that is known to have not existed. However, a conceptual synchronized and flattened trilobite germband – as presented for other taxa – would not be significantly different from the one presented for crown-group mandibulates, with the exception of there being only one antennal limb bud, the homologues of the second antenna and mandible would be structurally indistinguishable from trunk limbs, and specific differences in tagmatization. The embryonic origin of the typical trilobed organization of the trilobite body is unclear.

### Crown group chelicerata

**Fig. 8:**
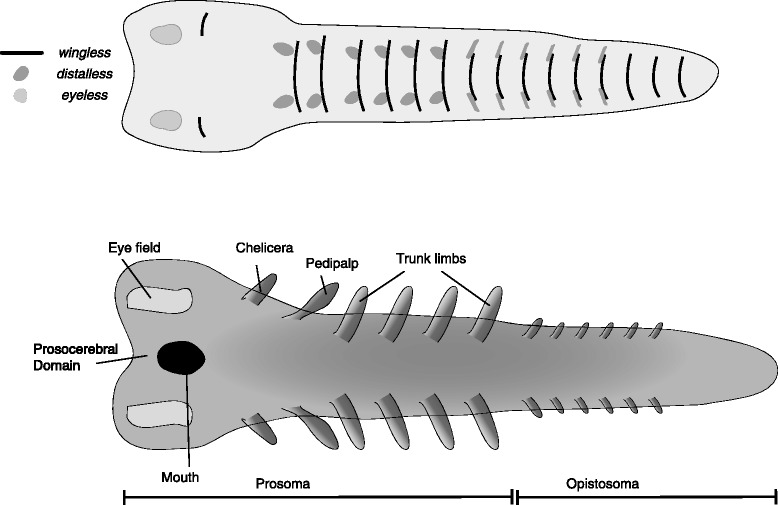
Reconstructed germband of the common ancestor of crown group Chelicerata, based on several extant taxa. The upper panel represents an early stage germband with the expression of select marker genes mapped onto it. The lower panel represents a late stage germband, annotated with the adult fate of the main embryonic features. The origin of the eyes has not been reconstructed in detail, and a broad “eye field” is shown instead. See [82] for a recent detailed description of eye development in spiders. Note the distinction between the prosoma and opisthosoma

The reconstruction of the germband of the hypothetical common ancestor of chelicerates (Fig. 8) is based on embryos of extant chelicerates, mostly the spider *Parasteatoda tepidariorum* [81, 82] and the harvestman *Phalangium opilio* [83, 84]*,* with some details added from the horseshoe crab *Limulus polyphemus* [85, 86]. I did not consider sea spiders (Pycnogonida), since they are believed to be highly derived morphologically. The head lobes are much smaller than those of extant mandibulates, and only include the protocerebral (ocular) and deutocerebral (cheliceral) segments. These are followed by the pedipalpal segment and four segments bearing walking limbs. All of these segments together form the prosoma. Additional segments follow, forming the opisthosoma. I have drawn 9 such segments, following the situation in spiders, though the ancestral number may be higher (e.g., as in scorpions). These segments are limbless in terrestrial chelicerates, though aquatic chelicerates (Xiphosura) have narrow, fused appendages forming the operculum and book lungs and anterior unfused chilaria. In the reconstruction I have drawn rudimentary limb buds in the anterior opisthosomal segments of late germband, and narrow *distalless* domains, as seen in *L. polyphemus.*

Dorsal closure does not include a “bend and zipper” movement as in some mandibulates [78], leaving the anterior limbs and the eye rudiments antero-ventral, rather than dorsal. In later development there is limited folding of the head lobes during dorsal closure, bringing the mouth to a sub-terminal position [81].

### Megacheirans

**Fig. 9:**
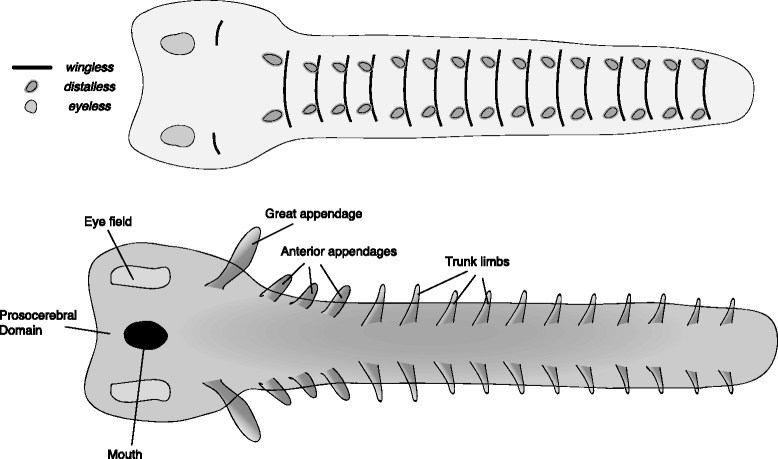
Reconstructed germband of Megacheira, based mostly on *Leanchoilia* spp. The upper panel represents an early stage germband with the expression of select marker genes mapped onto it. The lower panel represents a late stage germband, annotated with the adult fate of the main embryonic features

The reconstructed megacheiran embryo (Fig. 9) is based on data from several species, mostly *Leanchoilia* [9] and *Yohoia* [12], although the specifics of the figured reconstruction are based on *Leanchoilia.* Since the phylogenetic hypothesis used here sees Megacheira as a sister group to Chelicerata, similarities between the two groups are seen as synapomorphic. Thus, the reconstruction also draws inferences from the development of the chelicerates mentioned above, *P. tepidariorum* [81, 82], *P. opilio* [83, 84] and *L. polyphemus* [85, 86]

The head lobes encompass only two segments: the protocerebral segment and the deutocerebral segment. The protocerebral segment anlage contains the eye fields and the prosocerebral domain. The deutocerebral segment contains an extended raptorial appendage – the great appendage (GA). Posterior to the head lobes are three additional segments bearing small feeding appendage, followed by a trunk with a variable number of locomotory (swimming) appendages. The head lobes grow to encompass the three post GA segments, forming the cephalic shield.

While I have identified the GA as belonging to the deutocerebral segment and reconstructed the embryo accordingly, there is still some debate on this question. The identification of the megacheiran great appendage with the deutocerebral appendage and chelicerae is based both on positional homology and on a transformation series between fossil great appendages, through basal chelicerates to Euchelicerata [9, 12, 87]. In addition, preserved neural tissue in *Alalcomenaeus cambricus* indicates deutocerebral innervation of the great appendage [14]. Conversely, a few authors [8, 48] identify the GA as tritocerebral, and posit a deutocerebral antenniform appendage. This identification is apparently based on homologizing the SPA of fuxianhuids with the megacheiran GA. This assumption is unsupported by the reconstructed head morphology of most megacheiran fossils. The “antennate megacheiran” *Fortiforceps foliosa* is reported to have a deutocerebral antenniform appendage [50] but the quality of the material is poor, and this interpretation has been questioned. A tritocerebral identity of the GA would be consistent with a more basal phylogenetic position of Megacheira, as suggested by Legg et al.[8].

Posterior to the head lobes are three smaller limb-bearing segments, which seem to have a feeding role. These are followed by a series of trunk segments – 11 in the case of *Leanchoilia*. The head shield in the adult covers the three feeding segments. I assume homology of this head shield to that in the stem group taxa described above, and posit its origin in the anterior lobes, as in those cases. However, I only put the rudiments of the eye and GA within the head lobes, following the organization of the anterior germband in crown group chelicerates.

### Direct vs. indirect development

This entire exercise in embryonic reconstruction has implicitly assumed that all of the discussed organisms had direct development. In reality, there are very little data to indicate whether stem group arthropods had direct or indirect development. It is often assumed that the plesiomorphic state for arthropods is that of indirect development through a “head larva” with 3 or 4 segments. This is based on the existence of a head larvae in a fossil pycnogonid, of nauplii in basal mandibulates (many crustaceans), and on the extensive fossil evidence of trilobites and certain Orsten taxa, which also hatched as a head larva [88]. Conversely, the arthropod sister groups, Tardigrada and Onychophora exhibit direct development in all species.

The only direct evidence of larval forms in fossil arthropods (aside from trilobites) comes from an Early Cambrian megacheiran larva [13], which suggests a niche-differentiated larval form, but with a full complement of segments. Other megacheirans also show an ontogenetic series that suggests direct development through a series of adult-like larval instars with no dramatic metamorphosis [12].

Perhaps the most striking fact about larval forms of stem group arthropods is that they are never found. This is in contrast with larval forms of basal mandibulates, which are found abundantly in Orsten type assemblages [77]. While absence of evidence does not constitute evidence of absence, the fact that no larval forms of stem group taxa are found in numerous Orsten-like deposits, at times and in habitats where these taxa are known to have been present, and crown-group arthropod larvae are known (e.g., *Wujicaris* [89]), is suggestive of most of them having direct development, as seen in megacheirans. Indirect development probably evolved at the base of Mandibulata, although it could have evolved several times convergently in this group.

### Major transitions in the evolution of arthropod anterior structures

**Fig. 10:**
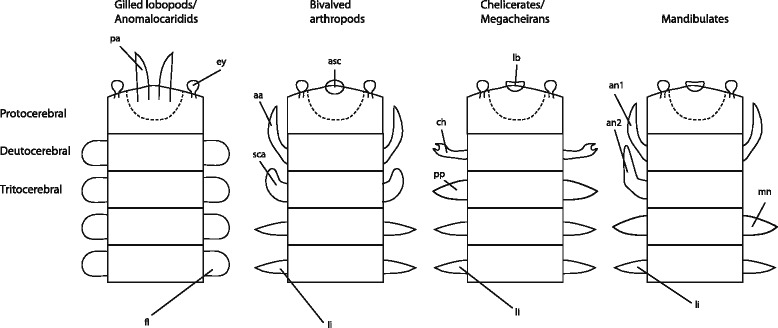
A schematic summary of the identity of anterior segments and their appendages in some of the taxa discussed, modified from [15]. The four illustrations are lined up with homologous segments at the same level. The anterior three are named on the left. The dashed line indicates the border between the prosocerebral domain and the archicerebral domain within the protocerebrum. Abbreviations: aa- antenniform appendage; an1- first antenna (crustacean antennule); an2- second antenna; asc- anterior sclerite; ch- chelicera; ey- eye; fl- flap (includes lobopod limb in *Kerygmachela*); lb- labrum (hypostome/labrum complex); li- limb (unspecialized walking or swimming limb); mn- mandible; pa- Primary antenna (raptorial limb); pp- pedipalp; sca- sub-chelate appendage

Focusing on the germband stage, as I have done here, indicates a series of major character transitions in the anterior part of the body throughout the evolution of the arthropod body plan (Fig. 10). The plesiomorphic condition was of a single protocerebral appendage. This appendage developed articulation before the splitting off of the anomalocaridids. It constituted the only differentiated anterior structure up to and including the Radiodonta.

A combination of major transitions took place (presumably rapidly) at the border between the lower stem and upper stem (Fig. 1). These include the appearance of the head lobes (=carapace and head shield), the reduction of the protocerebral appendage, and the appearance of two novel anterior appendages: a deutocerebral antenniform appendage and a tritocerebral subchelate appendage.

Within the crown group arthropods two independent series of transitions took place in the mandibulate lineage, and in the chelicerate lineage. The mandibulate lineage saw the differentiation of three trunk segments as accessory feeding appendages, leading to the complex six-segmented head with the novel feeding limbs situated ventrally, and the mouth pushed posteriorly relative to its ancestral position. In derived lineages, this was coupled with the evolution of the “bend and zipper” process of head capsule formation [78] led to the sense organs being situated dorsally,

The chelicerate lineage saw the deutocerebral appendage transformed from an antenniform appendage to a raptorial appendage, which was gradually transformed into a chelicera. The tritocerebral appendage retained its identity but underwent lineage specific transformation within crown group chelicerates to give rise to the diverse pedipalp structures found there. In the megacheirans this appendage lost its specific identity and transformed into a small appendage under the head shield, which may have had a role (together with three similar appendage pairs) as an accessory feeding appendage.

The anteriormost structure in the arthropod body plan – the ocular/protocerebral segment – has received a great deal of attention and has been the subject of intense debate in the last few years. This debate has been summarized succinctly in the Supplementary Discussion section in Ortega-Hernández (2015) [53]. The debate mostly revolves around the question of whether the protocerebral segment is a single segment or two segments. I see this as a mostly semantic discussion, and follow the suggestion that the ocular/protocerebral region should be seen as a complex structure [15], which is not a true serial homolog of more posterior segments. It is different from all other segments in its embryological structure (in extant arthropods), which includes both a median domain and a split lateral domain. It is also different in bearing two appendicular structures: the eyes (primitively stalked) and the protocerebral appendage (which transforms to the labrum), and possibly in having two pairs of ganglia [43].

### Major transitions in the evolution of arthropod posterior structures

The major transitions seen in the posterior germband mostly have to do with the gradual change from lobopod limbs to arthropodized limbs. Recent discoveries and analyses of anomalocaridid limbs suggest that this change took place via an intermediate stage of paired lateral flaps [19, 29]. However, this idea is preliminary and it is not clear how it could have occurred from a developmental perspective. The transition in limb morphology also included a recruitment of the jointed limb patterning network, probably involving Notch signaling and members of the *oddskipped* family [90, 91], as well as *extradenticle, dachshund* and *nubbin* [92]. This network was originally only present in the protocerebral appendage and was recruited to the development of all trunk limbs in the transition between the lower and upper stem group.

A second type of developmental transition in the evolution of the arthropod body plan was the evolution of tagmatization. Most stem group arthropods have homonomous or nearly homonomous limbs. There are few cases where the appendages immediately posterior to the head segments are smaller or slightly differentiated, and there are cases of individual specialized limbs [93]. However, clear differentiation between limb types in different regions of the trunk is only found in crown group arthropods. Similarly, groups of posterior segments that lack appendages entirely are also found only in the crown group and in the closely related fuxianhuiids. It is not unreasonable to assume that the evolution of limb differentiation is what allowed crown arthropods to out-compete other contemporaneous taxa and to emerge as the leading group.

### Upper and lower stem group arthropods

The analysis of major transitions in the evolution of the arthropod body plan indicates that there is a concentration of events in the transition between the lower stem and the upper stem group. The distinction between the two groups was recently stressed by Ortega-Hernández [30], who also pointed out a significant number of synapomorphies supporting the monophyly of the upper stem group. As indicated above, many of these synapomorphies represent significant embryonic novelties, including recruitment of whole gene regulatory networks to novel positions, appearance of novel embryonic structures, evolution of novel appendages and novel appendage morphologies and reduction of existing structures. Clearly there is a great deal of phylogenetic history and morphological evolution condensed within this one node, and it is of prime importance to try and identify fossils that can break up this node and indicate the sequence in which these numerous changes took place.

### The chelicerate question

The chelicerates are traditionally portrayed as sister group to all other extant arthropods and are therefore sometimes viewed by implication as being primitive. Seen in the context of arthropod evolutionary history, this interpretation is clearly wrong. The simple head (or lack of a head) in chelicerates should be seen as a secondary simplification, and a loss of differentiated anterior structures, assuming the current phylogenetic hypothesis (Fig. 1) is indeed true. Placing the megacheirans as sister to the chelicerates helps identify the process through which this simplification occurred. However, it is also possible that the chelicerate-specific anterior morphology is even more ancient. Given the similarities between the fuxianhuiid and mandibulate body plans – most notably in the existence of two specialized appendages within the head lobes, and the gradual evolution of specialized post-oral appendages – the split between mandibulates and chelicerates might be much deeper than presented in any published phylogenies (the most recent estimates put the timing of this split at late Ediacaran [7]). It is possible that the basal position of megacheirans is correct, and it is in fact the chelicerates that are placed too high in the tree. If we were to place the chelicerates as a sister group to basally positioned Megacheira, this would bring most of the upper-stem taxa into the crown group, prompting a significant re-thinking of arthropod evolution.

### An embryological perspective of the arthropod fossil record

The main objective of this paper is to provide a different perspective to that represented by most analyses of the early evolution of arthropods. My analysis strays significantly from the conventional cladistics-based approach. Rather than aiming to increase the number of characters and taxa used, and treating all characters as essentially equivalent, I have focused on representative taxa only, and chosen a small number of characters that can be seen at the germband stage of embryonic development. My inherent assumption is that characters present at these stages are fundamental to the body plan, less labile, and thus the changes they undergo throughout evolution represent the major steps in the evolution of the body plan.

I share the sentiments raised by Haug et al. [12], regarding the importance of a-posteriori analysis of phylogenetic trees. Similar doubts about the potential pitfalls in large phylogenetic datasets have been raised in the past by Jenner [94, 95]. I have based my analysis on a previously published phylogeny, generated using a large cladistic dataset [8], but have taken the liberty of rejecting several aspects of this phylogeny where it is inconsistent with the proposed scenario of major evolutionary transitions in the body plan.

## Conclusions

In this paper, I have presented hypothetical reconstructions of embryos of fossil arthropods. These reconstructions suggest several novel homology hypotheses – e.g. the lower stem group head shield and head capsules in the crown group are all hypothesized to derive from the embryonic head lobes. The homology of anterior segments in different groups is resolved consistently. The transition between “lower-stem” and “upper-stem” arthropods is highlighted as a major transition with a concentration of novelties and innovations, suggesting a gap in the fossil record. A close relationship between chelicerates and megacheirans is supported by the embryonic reconstructions, and I suggest that the depth of the mandibulate-chelicerate split should be reexamined. Finally, I stress again that the reconstructions of the germbands of fossil arthropod taxa, which form the core of the current analysis, are meant to be seen as heuristic tools for tracing the evolution of the arthropod body plan, and should not be seen as definite. Hopefully, these can be used as a basis for modification and discussion, and can be used to trace the evolution of other structures and characters that were not included within the scope of this analysis. Mostly, it is hoped that this unusual perspective will be useful for students of arthropod evolution in conventional disciplines and will contribute to a better understanding of the many unresolved issues in the field.

## Methods

### Reconstructions

Each taxon includes two reconstructions. The first is an early germband, before limb buds first appear, but with segmental boundaries mapped (represented by *wingless* stripes) and sites of limb outgrowth (represented by *distalless* domains). The putative area from which the eyes will develop is plotted as a domain of *eyeless* expression. The second reconstruction is a late germband, with limb buds and other structures already visible. These later reconstructions are annotated, and constitute a fate map.

Crown group arthropod germbands are reconstructed based on extant arthropod germbands. Lower-stem groups are reconstructed using the onychophoran germband as a basis, with novel structures added to these. Upper-stem germbands are reconstructed through a series of hypothetical steps connecting the onychophoran-like germband with an arthropod-like germband. In positioning the anlagen of different structures on the germband, I have followed a rule of thumb in which more medially located structures in the germband end up ventral in the adult, and more laterally located structures end up dorsal, following dorsal closure and the transformation of the two-dimensional germband into a three-dimensional animal.

### Availability of supporting data

All supporting data are available in the article and in the cited references.
